# Laterality Influences Schooling Position in Rainbowfish, *Melanotaenia* *spp*

**DOI:** 10.1371/journal.pone.0080907

**Published:** 2013-11-15

**Authors:** Anne-Laurence Bibost, Culum Brown

**Affiliations:** Department of Biological Sciences, Macquarie University, Sydney, Australia; The Australian National University, Australia

## Abstract

Cerebral lateralization is a widespread trait among animals, is often manifested as side biases in behaviour (laterality) and has been suggested to provide fitness benefits. Here we examined the influence of laterality on the organisation of fish schools using rainbowfish (*Melanotaenia* *spp*) as model species. The pattern and strength of laterality for each individual was determined by examining eye preferences whilst examining their reflection in a mirror. Schools of four fish of known laterality were then created and the preferred position for each fish within the school was repeatedly observed in a flume. Fish which showed right eye preferences in the mirror test preferentially adopted a position on the left side of the school. Conversely, fish that showed left eye preferences in the mirror test or where non-lateralised preferentially adopted a position slightly to the right side of the school. However, this general pattern varied depending on the species and sex of the school. Our results strongly implicate individual laterality in the geometry of school formation.

##  Introduction

Many vertebrates and invertebrates show a preferential use of one side of their body over the other; a phenomenon known as laterality [1-3]. Laterality stems from cerebral lateralization, whereby specific types of information are preferentially processed in one hemisphere of the brain. With an ever increasing number of vertebrate species examined it is becoming clear that laterality is a trait shared among all vertebrates and likely originated in a common fish-like ancestor [4]. 

It is now widely accepted that brain lateralization conveys both costs and benefits while performing certain tasks and that it can have fitness consequences for animals in their natural environment [5]. Previous studies have demonstrated that strongly lateralized animals perform better than non-lateralized animals in a variety of contexts. For example, Magat and Brown [6] found that strongly lateralized parrots were faster at learning a complex task than non-lateralized parrots. In addition, strongly lateralized parrots and domestic chickens were faster in discriminating between pebbles and grains than non-lateralized individuals [6,7]. Strongly lateralized quail are also better at distinguishing familiar from unfamiliar conspecifics than non-lateralized individuals [8]. Moreover, brain lateralization is suggested to enhance simultaneous task performance such as foraging whilst also looking out for predators in a range of taxa [9,10]. The costs of laterality are various and context specific. For example, strongly lateralized animals often have difficulty in solving spatial tasks because their inherent turn bias can be difficult to overcome [11]. Similarly strongly lateralised individuals perform relatively poorly when they have to compare similar information in each visual hemifield [12]. 

The observed pattern of laterality across species, and particularly the variation within species, is likely shaped by natural selection to suit contemporary ecological and social conditions [6,13-15]. Large bodied parrots that use extractive foraging techniques tend to be strongly lateralized whereas small bodied species that graze on grass seeds and nectar are non-lateralized [13]. In addition, the pattern of lateralization varies between populations subject to differential predation pressure. Fish from high predation regions are more strongly lateralized compared to fish from low predation regions and their pattern of laterality also differs [16]. It has been argued that fish from high predation locations, or those that readily rely on schooling, show enhanced laterality so that they can keep track of their shoal mates and other stimuli simultaneously [16-18]. 

Laterality has been extensively studied using fish as model organisms (reviewed by [5]). A large number of fish species form schools (a cohesive group of fish that swim in polarised and synchronize manner) or shoals (a loose social aggregation of fish) [19]. Group cohesion provides advantages by enhancing foraging success and anti-predator behaviours [20]. It is easy to imagine how such finely tuned manoeuvres could be influenced by laterality. One might predict, for example, that the stability and the cohesion of a fish school are preserved if all the fishes tend to swim in the same direction. Alternatively, perhaps schools are best comprised of a range of lateralized individuals that prefer to take up different locations within the school. It is possible that fish with either a right eye or a left eye bias for viewing conspecifics would be positioned on the left and right side of the school respectively. This would allow lateralized fish to simultaneously gather information about their school mates in one hemifield and other key stimuli in the contra-lateral hemifield (eg predators or prey). In theory, this would enable them to perform more efficient anti-predator or foraging behaviour due to their ability to process the information more quickly in the appropriate hemisphere. Under this scenario one would expect a school made up of a mixture of literalities and there may be some degree of frequency dependent selection acting on lateralized phenotypes in these highly social contexts [21]. Previous work has found that pairs of lateralized fish, *Girardinus falcatus*, are more cohesive and synchronised while schooling in a novel environment than pairs composed of weakly lateralized fish [22]. In a group comprised of three fish, non-lateralized individuals tended to be located at the periphery of the school. Conversely, lateralized individuals were located in the centre of the school where predation risk and the energy expenditure for swimming is lower [22,23]. Thus, differences in laterality between individuals do seem to be an important factor in the organisation of a fish school.

Here we examined the relationship between individual lateralization and the position fish adopted within a school comprised of four individuals. Our model species were members of the genus *Melanotatenia*, facultative schooling fish endemic to Australia. We chose two species which differ in their tendency to school. For each of these species, both wild and captive-reared populations were tested. Previous studies have shown that captive rainbowfish tend to lose their shoaling behaviour [24]. Thus our comparison potentially encompassed a wide range of schooling tendencies. We hypothesised that if the position adopted by fish is independent of its pattern of laterality, then we expect individual fish to be position randomly in the school. Alternatively, if laterality influences schooling position, strongly lateralized fish are expected to position themselves in specific locations within the school. More specifically, we expected fish to take up positions in schools that correspond to their visual hemifield preferences for observing conspecifics.

## Methods

### Subjects

Captive and wild *Melanotaenia* species (*M. duboulayi and M. nigrans*) were used as test subjects. For this study, we collected 16 males and 16 females from each of the four populations. Captive-reared *M. nigrans* were obtained from a commercial supplier (Aquagreen, Darwin) and captive-reared *M. duboulayi* were obtained from the Environmental Protection Agency (EPA, Sydney). The captive-reared *M. duboulayi* have been bred in captivity for multiple generations [24]. Throughout this time they were housed in large, featureless aquaria with a gravel substrate. The captive *M. nigrans*, in contrast, were reared in large outdoor ponds. Wild *M. nigrans* were collected from Rapid Creek, Northern Territory (12°23 S, 130° 52 E) and wild *M. duboulayi* were collected from Orara River, New South Wales (30°15 S, 150°00 E). Both populations were collected using a 4m seine net with 10mm mesh. While wild both populations are exposed to a number of predators, the Rapid Creek population tend to be exposed more sporadically as dictated by tidal regimes. Theoretical models predict that the sporadic and unpredictable exposure to predators offers the highest risk to prey [25] and our observations of these populations show that wild *M. nigrans* has the strongest schooling tendency. All fish were transported to Macquarie University, Sydney, Australia where they were housed separately. Housing consisted of large aquaria (110×30×30cm) illuminated by overhead fluorescent lights. The water temperature was maintained at 26°C and the pH was approximately neutral. The fish ranged in size from 30-40 mm depending on species, origin and sex.

Wild fish were tested after two months of life in captivity to eliminate handling stress. All fish used in this experiment were adults, ranging from one to three years of age. To aid individual recognition, all fish were tagged with a Visible Elastomer Implant (VIE) a week before the experiment. Tagging rainbowfish using VIE has no effect on behaviour [26] and all fish showed full and rapid recovery.

### Laterality test

**Figure 1:**
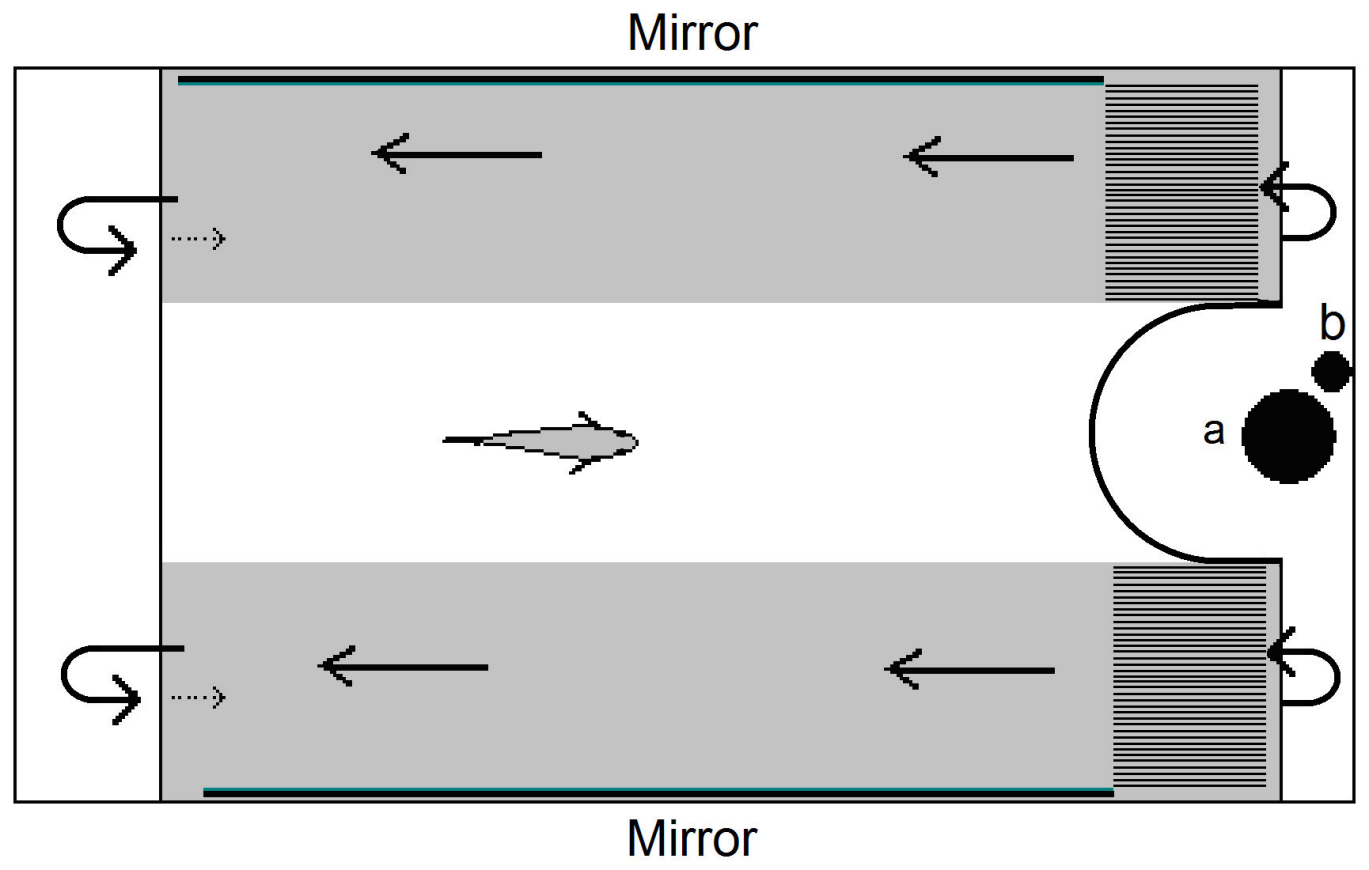
A diagram of the laterality test. Subjects swim against the water flow and maintain station with their mirror image on the left or right side of the apparatus. Arrows indicate water flow in the experimental area (solid lines) and the section below (broken lines). Grey areas are regions of laminar flow generated by banks of straws. The central area is an area of low flow generated by a baffle. The location of the (a) pump and (b) heater are shown.

Mirror tests are often used to examine laterality in fishes [27]. Here we used a flume measuring 110cm × 30cm × 30cm because rainbowfish tend to school best in flowing water (Figure 1). A mirror was placed on either side of the test compartment to simulate the presence of shoal mates using the subject’s reflection. The apparatus was designed with two levels with a pump on the bottom level generating laminar flow in the test compartment on the upper level. The flow generated by the pump (32L/min) encouraged the test subject to swim against the water current and thus maintain station with their reflection. The flow was directed along the left and right sides of the test compartment through two banks of straws (20cm long) with a baffle in the middle creating a zone of low flow in the centre and laminar flow to either side. The nature of the flow was examined using dye tests which revealed that the laminar flow area extended approximately 10cm into either side of the flume. During the observation, the flume was isolated from the rest of the laboratory using curtains and observations were made via a web-camera suspended above the apparatus.

Subjects were removed from their home tank and carefully transferred to the flume for a 10 min observation period. Subjects were expected to school with a conspecific (i.e., their mirror image) on the left or right side of the flume, depending on their eye preference. The position of the subject with respect to the mirror was recorded every 10 s. Fish swimming within two body lengths of the mirror on the left or right hand side were scored as preferring the right or left eye to view conspecifics respectively. Fish located in the centre of the flume were scored as non-lateralized. At the end of the 10 min observation period, laterality index was calculated according to the formulae: time spent on the left side / (time spent on left and right sides). 

### Schooling test

Based on the outcome of the laterality tests, we created schools comprised of two left and two right lateralized individuals of the same sex. Rainbowfish are frequently observed in small schools in the wild [28]. All individuals within the school were size-matched and were familiar with one another. 

**Figure 2:**
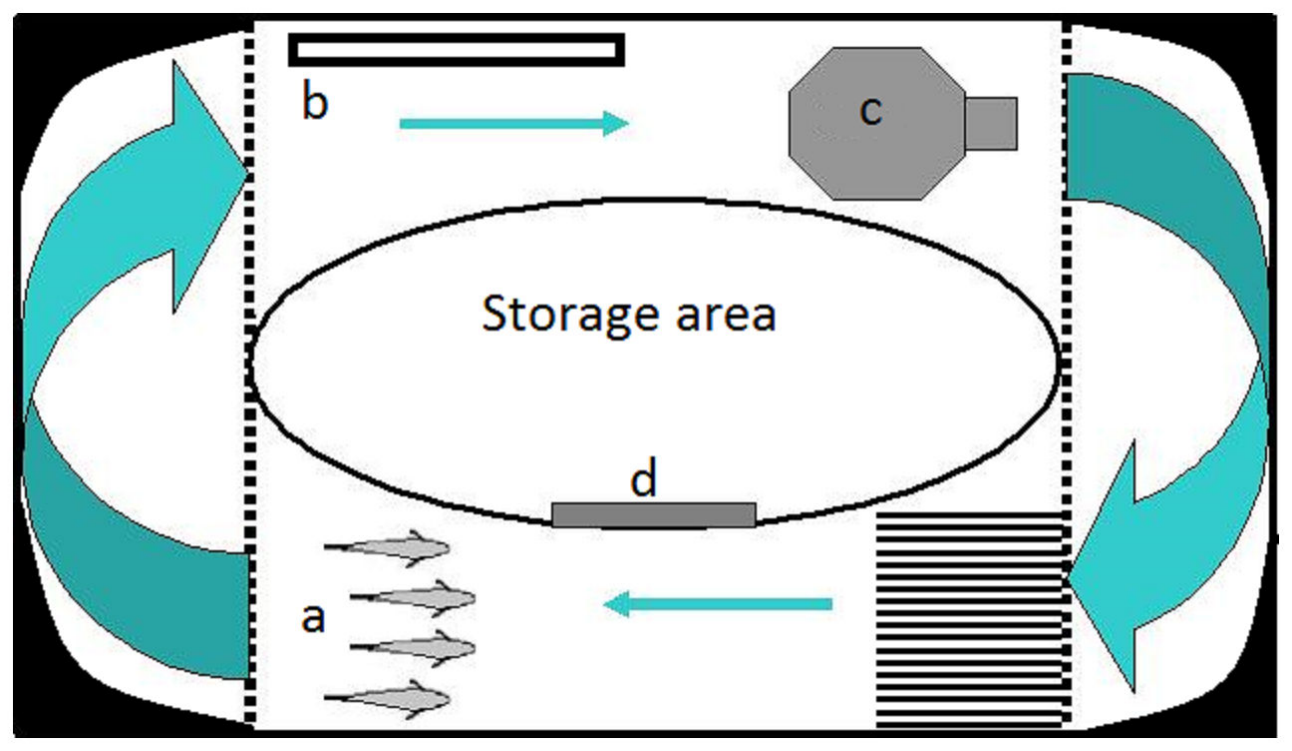
A diagram of the flume in which the preferred location of each fish within a school was tested. Arrows indicate the water flow and the broken lines indicate the mesh barriers confining subjects to the test area. The position of the (a) subjects, (b) heater, (c) pump and (d) trap door are illustrated.

A circular flume was inserted into a tank measuring 95cm × 50cm × 22cm to examine the position adopted by each fish within the school (Figure 2). A storage room with a trap door was located in the middle of the arena. The water was circulated around the periphery with a 32L/min pump to encourage the school to swim against the water current. Fish were confined to the observation zone by mesh barriers. Laminer flow was generated by a bank of straws located immediately upstream just in front of the observation zone. The height of the water was 10cm, gravel was used as substrate, the water temperature was 26°C and the pH was maintained at 7. The entire flume was illuminated by overhead fluorescent lights and an additional blue light to make the VIE tags fluoresce. A high definition wireless camera was positioned over the top of the schooling zone.

Each school was introduced to the store room for 15 min to allow the fish to adjust to their surroundings. The trapdoor was then lifted remotely to provide access to the arena. Once all members of the school had left the storage room the trapdoor was closed and recording began. Observations of the position of individuals within the school were recorded every time the subjects formed a tight school. The position of the each fish was noted at a single time point 10 s into the schooling period. Here we defined the school when all individuals were within 2 body lengths of one another and swimming in a polarized, coordinated fashion [17]. The position of each fish in each group was noted by two observers for twenty schooling events. Each observer watched two fish and recorded their position of the fish in each school in real time while watching the footage in real time on a large video screen. The footage was also recorded via digital camera as a backup but was rarely consulted. The position for each individual in the school was tabulated by allocating a value as follows: 1 = extreme right; 2 = centre right; 3 = centre left; and 4 = extreme left. The preferred (most commonly adopted) position of each individual was calculated over the 20 schools. In only a single case did a fish show an ambiguous choice, with an equal preference for position 2 and 3. This individual was assigned a 2 in the analysis though it made no difference to the overall results.

Preferred school position was analysed using an ANOVA where species, rearing environment, sex and laterality score were considered as factors. In the first analysis, fish which spent > 50% of the time on the right side of the flume during the laterality test were categorised as right lateralized, and fish that spent < 50% of the time on the right hand side of the flume were categorised as left lateralized. In the second analysis, fish were divided into strongly left or right biased fish (<80% on the left of right side of the flume respectively) with the remaining fish labelled as non-lateralized. This second analysis was relatively limited in power subject to the laterality distribution of the subjects.

We also specifically examined if the fish preferred peripheral or central positions using logistic generalized linear model. In this context we used the log_10_ of the raw laterality score as a continuous variable, species, rearing environment and sex were independent variables. 

### Permits

The work in this paper was authorised by The Macquarie University Animal Ethics Committee (ARA; 2010/028). Fish were collected from the wild under fisheries permit P08/0010-3.0.

##  Results

**Figure 3:**
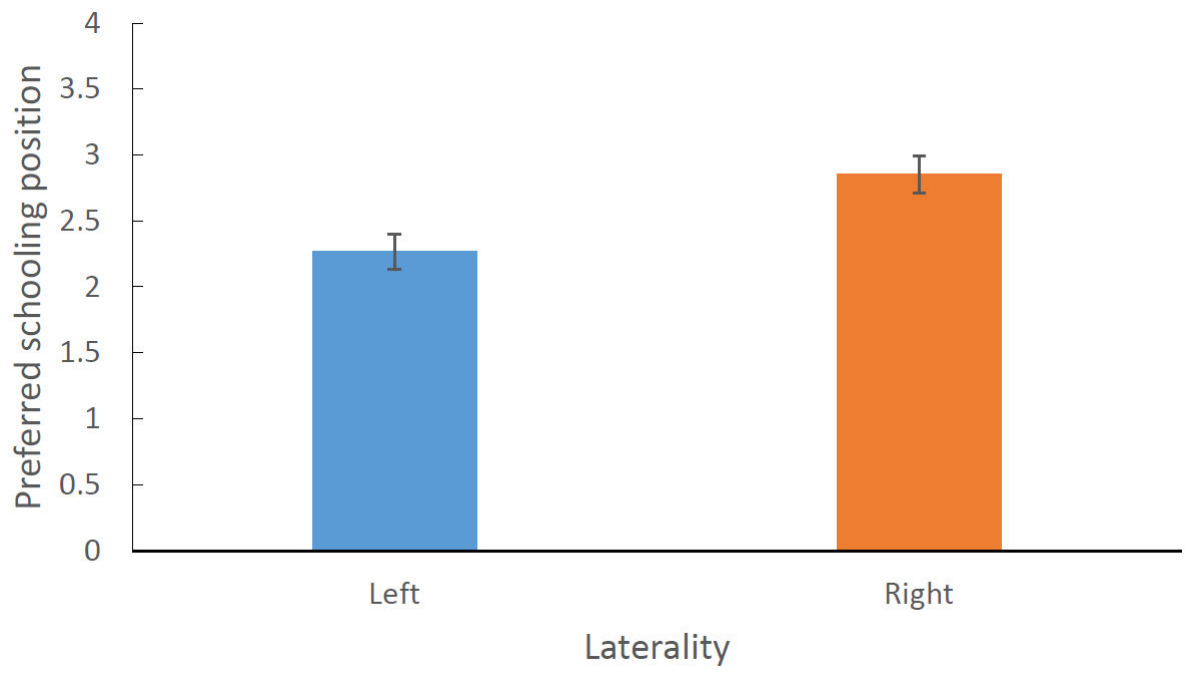
The mean (±SE) preferred schooling position for left and right lateralized fish. Scores greater than 2.5 represent a preference for the left side of the school.

**Figure 4:**
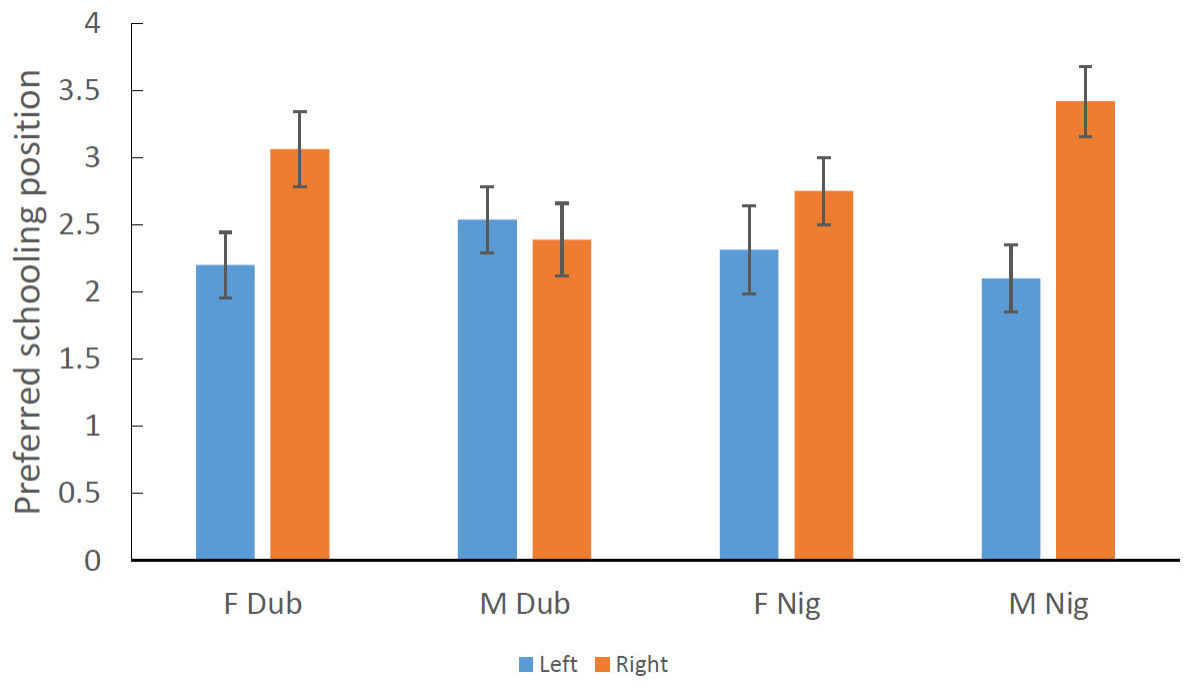
The mean (±SE) preferred schooling position for left and right lateralized *M. duboulayi* and *M. nigrans*. F = female, M = male. Scores > 2.5 represent a preference for the left side of the school.

Our models showed that rearing environment (captive or wild) had no main effect and no interaction with any other terms and was thus removed from the models. The simplified model using a dichotomous laterality index showed that laterality had a significant effect on schooling position such that right lateralised fish tended to take up positions on the left side of the school and visa-versa (F _1, 119_ = 10.224, P = 0.002; Figure 3). We also found a three-way interaction between species, sex and handedness (F _1, 119_ = 5.978, P = 0.016; Figure 4). Post-hoc tests revealed that female *M. duboulayi* and male *M.nigrans* both showed significant effects of laterality on their preferred schooling position (Fisher’s PLSD: P = 0.028 & 0.002 respectively). Female *M. nigrans* showed a similar trend but it was not significant, while male *M. duboulayi* showed no relationship between laterality and school position.

**Figure 5:**
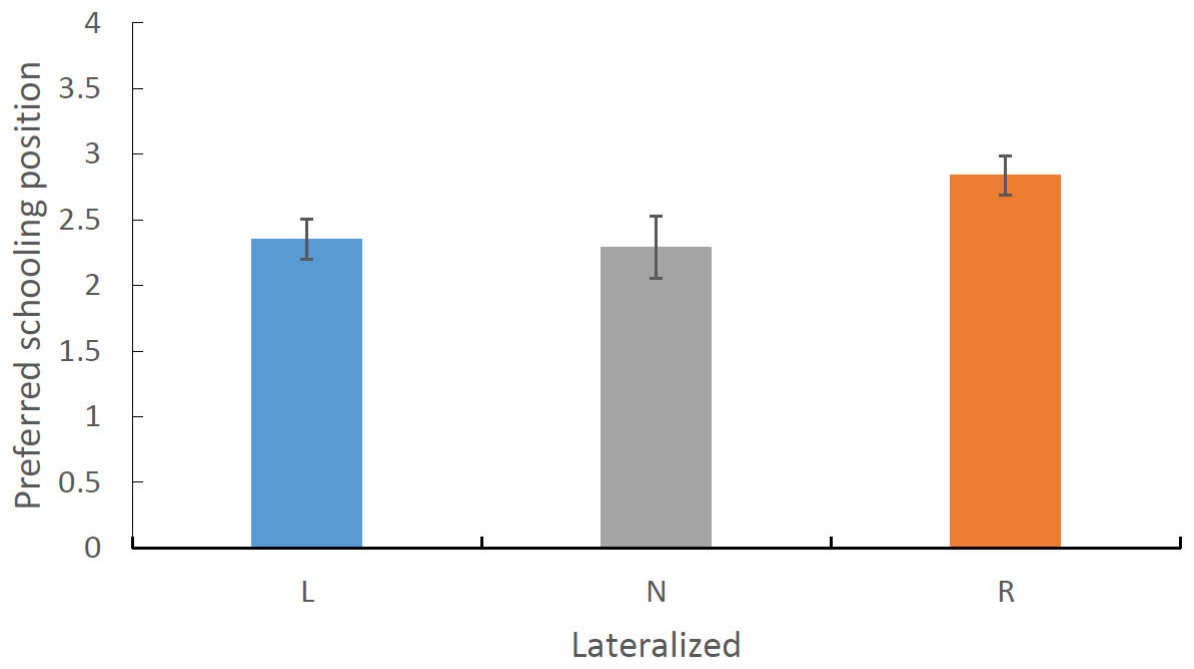
The mean (±SE) preferred schooling position for strongly left, non and strongly right lateralized rainbowfish. Scores greater than 2.5 represent a preference for the left side of the school.

The second analysis of the data using the three laterality categories only found a significant main effect of laterality on the preferred schooling position (F_1, 115_ = 3.673, P = 0.028) such that strongly left biased fish took up positions on the right side of the school, non-lateralized and right biased fish preferred positions just to the left side of the school (Figure 5). No other effects where found.

**Figure 6:**
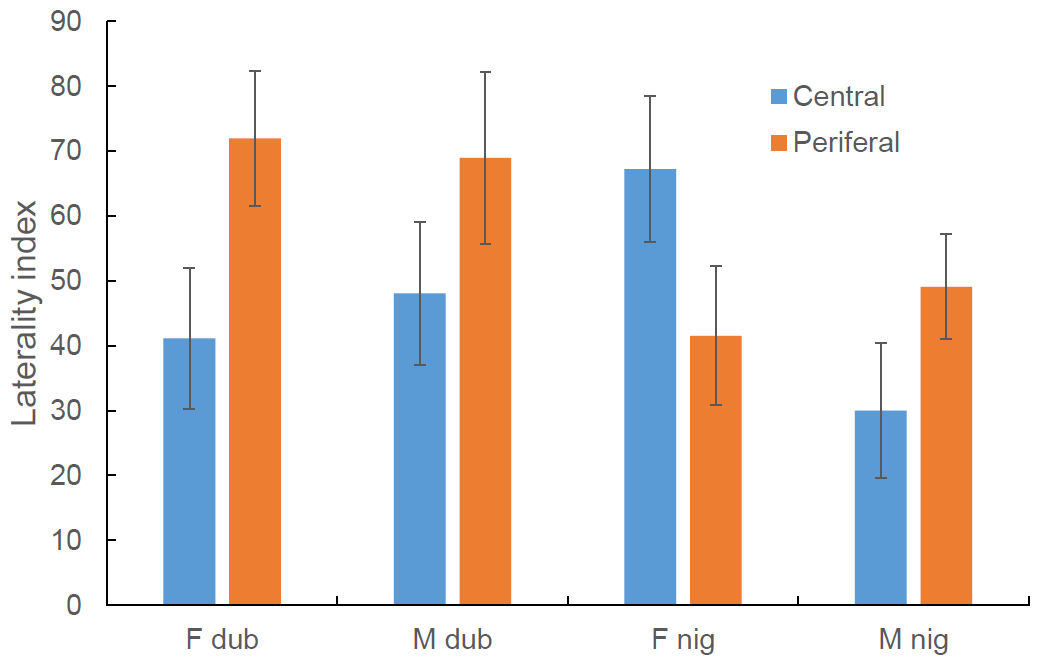
The mean (±SE) laterality index of male (M) and female (F) rainbowfish from two species; *M. duboulayi* and *nigrans* taking up central or peripheral locations in a school of four fish. Note the statistics are based on logistic models using log_10_ (laterality index).

Our logistic models examining the dichotomous preference for the peripheral or central positions found a significant main effect of laterality index (Wald Chi^2^ = 4.392, P = 0.036) whereby peripheral fish had a stronger right eye bias. A significant interaction between sex, species and laterality index (Wald Chi^2^ = 6.571 P = 0.010) was also revealed such that all fish followed the general pattern except female *M. nigrans* which showed the opposite pattern (Figure 6). As with the previous models, rearing environment had no influence on the model and was subsequently removed. 

## Discussion

This study provides evidence of a relationship between laterality and the position fish adopt within a school. Generally speaking, fish tended to choose positions within the school that matched their laterality scores but there were intriguing variations between species and sexes. Contrary to expectations, rearing environment had no influence on the relationship. Generally fish that showed a left eye bias for observing their mirror image adopted a position on the right side of the school, where as fish that showed a right eye bias for their mirror image tend to be found just to the left of the centre of the school (Figure 3). These results support previous data that shows that laterality influences schooling behaviour, a highly important fitness-related implications for foraging and anti-predator behaviour [29].

It is well documented that the position adopted by a fish when swimming within a school is influenced by a range of factors, including the internal motivational state (eg. level of hunger), hydrodynamics and predator avoidance strategies [29–31]. Moreover, positions within a school have different costs and benefits associated with them [32]. For example, peripheral positions may enhance foraging opportunities, but they are also more vulnerable to predation [33]. Individuals within populations vary in their laterality scores and the present study suggests that each fish positions itself within the school accordingly. Individuals that were more left lateralized when viewing their mirror image were found in positions at the periphery of the school keeping the majority of their shoal mates within their preferred visual field. It is highly likely that this school position is the product of an active choice on the behalf of the fish which compete for their preferred positions within the school in a highly dynamic fashion and is dependent on the laterality scores of the other fish in the school. It may be that strongly lateralized fish (particularly right biased individuals) benefit from occupying in these positions but we have yet to conduct tests which involve predator or prey detection in a schooling context. 

To date only a single study has examined the influence of laterality on schooling position. Bisazza and Dadda [34] observed that lateralized fish tended to be positioned in the centre of the school of three fish while non-lateralized fish were position on the periphery. Here the majority of strongly lateralized fish were found in peripheral positions with the exception of female *M. nigrans* which displayed the reverse pattern. Interestingly, even the male *M. nigrans* occupying peripheral positions within the school were more left biased than those occupying central even though the former where generally non-lateralised while the latter tend to be left lateralized during the eye preference test. While the findings of Bisazza and Dadda [29] generally contrast with the present study, it should be noted that they used *Girardinus falcatus* from selected lines reared in a captive environment. Given the species and sex differences reported herein, caution must be applied when generalising across taxa. Nevertheless, the findings from both studies clearly show that laterality clearly influences schooling behaviour.

Lateralization in a group living context might be of particular benefit to species that rely on coordinated behavioural responses to increase their chance of survival [21,35]. Groups comprised of mixed laterality types may enhance schooling performance by enhanced cognition mediated via rapid, simultaneous cue processing [35]. The practical manifestation of this is the optimal organization of individuals for detecting predators and prey in a social group. When a predator approaches, for example, the left or right lateralized fish on the periphery of the school will detect and respond to it more efficiently than a non-lateralized fish. Once an escape response is initiated it would rapidly spread through the school via the chorus line effect [36]. Weakly lateralized fish in the centre of the group can effectively follow fish on either side of the shoal since they display no eye preferences. The whole group would thus be synchronized when moving, preventing any loss of fish during shoaling [35]. We suggest that a mix of lateralised phenotypes in a school might increase individual fitness during social interactions (a quasi-group-selectionist hypothesis). Ultimately, groups comprised of mixed laterality phenotypes allow individuals to obtain an optimal position within a school and this likely enhances school coordination and cohesion, thereby increasing individual fitness. This might explain why there is so much variability in laterality scores in natural fish populations [37].

There are notable exceptions to the general finding that laterality influences schooling position as indicated by the three-way interaction between laterality, sex and species. In *M. duboulayi*, females showed the strongest preferences for adopting a specific position within a shoal reflecting their laterality scores, while for *M. nigrans* it was the males. Firstly it is important to note that we expected *M. nigrans* to show the strongest preferences because they school more strongly. This is generally borne out by the fact that both sexes showed similar patterns and the males in particular showed very strong associations between eye preference and schooling position. In contrast, female *M. duboulayi* showed reasonably good associations, but the males showed none at all (Figure 4). There are a number of possible explanations for this observation but the most likely stems from the possible problems of testing laterality using mirror images. While our schools were comprised of familiar individuals, the laterality test relied on a mirror image which to the fish likely represents an unfamiliar individual (fish rarely see their reflections in the wild). Previous studies have shown that fish (and indeed other vertebrates) often engage the opposite hemisphere when observing unfamiliar individuals, probably because they represent a potential threat [27]. The extent to which each of these species and sexes tend to associate with familiar individuals is largely unknown, although previous tests have shown that female *M. duboulayi* do show strong preferences for familiar individuals irrespective of context [38]. There are also other differences between schooling with a mirror image and schooling with real conspecifics, not least of which is the multi-modal feedback each fish gets from the rest of the school mates. Of course there are real hydrodynamic benefits to be had when schooling with real conspecifics which are entirely lacking with mirror images. Feedback from real conspecifics is likely to be appropriate whereas the feedback from mirror images obviously lack this dynamic feedback. 

Secondly, there is clear evidence in the literature that laterality is affected by predation pressure. A comparison between 16 species of fish revealed that shoaling species were more often lateralized than solitary species [17]. Fish from high predation populations are also more strongly lateralized compared to fish from low predation populations [16–18]. Additionally, fish from high predation areas, where schooling behaviour is under intense selective pressure, form more cohesive schools [39-41]. Moreover, it has been hypothesised that laterality evolves as a result of social pressure to allow groups of animals to coordinate with each other and this is likely enhanced under high predation pressure particularly in a schooling context [21]. We hypothesised that *M. nigrans* would show a stronger relationship between laterality and the position adopted within the school because the predation pressure they face is likely higher than that of *M. duboulay*. This was largely borne out here. However, predation pressure and anti-predator responses also vary between sexes largely due to variations in life-history priorities [42]. Thus one might expect differences in laterality and schooling behaviour between species and sexes within species. 

To conclude, there is mounting evidence that fish show preferences for particular positions within the school depending on a range of factors, including laterality. However, the strength of the association between laterality and the position fish adopt in a school can vary between species, possibly depending on the extent to which they are obligated or facultative schoolers and between sexes due to variation in life history priorities. 
